# Lifestyle Factors and Visible Skin Aging in a Population of Japanese Elders

**DOI:** 10.2188/jea.JE20090031

**Published:** 2009-09-05

**Authors:** Keiko Asakura, Yuji Nishiwaki, Ai Milojevic, Takehiro Michikawa, Yuriko Kikuchi, Makiko Nakano, Satoko Iwasawa, Greg Hillebrand, Kukizo Miyamoto, Masaji Ono, Yoshihide Kinjo, Suminori Akiba, Toru Takebayashi

**Affiliations:** 1 Department of Preventive Medicine and Public Health, Keio University School of Medicine, Tokyo, Japan; 2 Procter & Gamble Japan, Kobe, Japan; 3 National Institute for Environmental Studies, Tsukuba, Ibaragi, Japan; 4 Okinawa Prefectural College of Nursing, Graduate Study in Health Nursing, Naha, Japan; 5 Department of Epidemiology and Preventive Medicine, Kagoshima University, Graduate School of Medical and Dental Sciences, Kagoshima, Japan

**Keywords:** skin aging, quantitative method, Japan, older adults, lifestyle

## Abstract

**Background:**

The number of studies that use objective and quantitative methods to evaluate facial skin aging in elderly people is extremely limited, especially in Japan. Therefore, in this cross-sectional study we attempted to characterize the condition of facial skin (hyperpigmentation, pores, texture, and wrinkling) in Japanese adults aged 65 years or older by using objective and quantitative imaging methods. In addition, we aimed to identify lifestyle factors significantly associated with these visible signs of aging.

**Methods:**

The study subjects were 802 community-dwelling Japanese men and women aged at least 65 years and living in the town of Kurabuchi (Takasaki City, Gunma Prefecture, Japan), a mountain community with a population of approximately 4800. The facial skin condition of subjects was assessed quantitatively using a standardized facial imaging system and subsequent computer image analysis. Lifestyle information was collected using a structured questionnaire. The association between skin condition and lifestyle factors was examined using multivariable regression analysis.

**Results:**

Among women, the mean values for facial texture, hyperpigmentation, and pores were generally lower than those among age-matched men. There was no significant difference between sexes in the severity of facial wrinkling. Older age was associated with worse skin condition among women only. After adjusting for age, smoking status and topical sun protection were significantly associated with skin condition among both men and women.

**Conclusions:**

Our study revealed significant differences between sexes in the severity of hyperpigmentation, texture, and pores, but not wrinkling. Smoking status and topical sun protection were significantly associated with signs of visible skin aging in this study population.

## INTRODUCTION

It is generally accepted that a youthful facial appearance correlates with prolonged survival, and that several environmental factors are associated with facial aging.^1^ Therefore, lifestyles which prevent facial skin aging are probably important in improving overall health. Although accurate measurements of facial skin attributes are required to examine the associations between skin condition and various factors, most methods are subjective, because they depend on visual grading by trained examiners.^2^^–^^4^ Several techniques for examining skin condition quantitatively have been attempted,^5^^–^^8^ including imaging methods.^9^ Battistutta et al described a technique of grading the surface topography of sun-exposed skin by using a silicone impression of the skin surface.^5^ This method was noninvasive, but the silicone impressions were visually assessed by 2 graders. Considerable skill and experience might be required of the graders to obtain accurate results. Another method was reported by Sandby-Moller et al.^6^^–^^8^ This method was based on the use of a skin reflectance meter, ultrasonography, or a fluorescence spectrometer. Only skin pigmentation and redness were measured, and specially trained technicians were sometimes required to perform the measurements, especially ultrasonography. Kollias et al reported a method using fluorescence spectroscopy.^9^ This method was capable of measuring the constitution and pigmentation of skin, but the relationship between other aspects of facial skin appearance and the measured values was sometimes ambiguous. Therefore, in the present study we attempted to use more straightforward, noninvasive, standardized digital imaging paired with objective and quantitative computer image analysis to assess important visible features of facial skin aging, namely, hyperpigmented macules (spots), pores, texture, and wrinkles.

It is thought that the features of skin aging differ between Asians and whites.^10^ For example, it has been shown that the appearance of skin aging differed between French and Chinese women.^11^ Although there are several studies of the association between skin condition and certain lifestyle factors among whites,^4^^,^^12^^,^^13^ the number of reports on Asian skin condition and its relation to lifestyle factors is limited.^14^^–^^17^ As for Japan, Yin et al examined the association between skin aging and 2 environmental factors—ultraviolet exposure and tobacco smoking—in a cross-sectional study, and found that both were associated with worse skin condition.^15^ However, their study included only 83 subjects. In addition, most studies on changes in skin condition with age involved subjects aged approximately 70 years or younger. Since the effects of both host and environmental factors on skin aging require years or decades to manifest clinically as visible signs, we decided to measure the skin condition of an older population. By doing so, the long-term effects of various factors would be more apparent.

Thus, in a population of community-dwelling Japanese elders, we measured facial skin parameters quantitatively using an objective method and examined the association between their skin condition and a wide range of lifestyle factors.

## METHODS

### Study population

The target population was the older residents of the town of Kurabuchi (Takasaki City, Gunma Prefecture, Japan), a mountain community with a population of approximately 4800. During 2005 and 2006, trained public health nurses administered home-visit health surveys using a structured questionnaire. All persons aged at least 65 years were included in the study, except those who were in a hospital or nursing home. Ultimately, 1446 persons were identified as eligible subjects for this study, and of those, 802 (341 males, 461 females) agreed to participate in the facial skin condition assessment study. These assessments were conducted at 8 town community centers over a 3-month period (April through June). As compared to the 1446 survey-only participants, the 802 skin assessment participants included a higher proportion of females (50% vs 57%, respectively) and a lower proportion of persons aged 80 years or older (37% vs 25%, respectively).

The study was approved by the Medical Ethics Committee of the School of Medicine, Keio University, Tokyo, Japan, and all subjects gave their written informed consent. No incentives were offered for participation.

### Image collection and analysis

Facial images were collected using a 1.3-megapixel CCD camera (Fuji DS330) equipped with a close-up lens mounted on a standardized illumination rig (6500°K high-frequency fluorescent white light source) fitted with head-positioning aids^18^^,^^19^ (Beauty Imaging System, [BIS], Procter & Gamble Japan, Kobe, Japan). The camera was white-balanced daily. Subjects were not allowed to wear makeup, jewelry, or glasses. Their skin was cleaned with a gentle facial make-up remover/cleanser 20 minutes before image capture. The subject’s clothing was covered by a black cape or draping. After positioning the subject’s face in front of the camera, an image of the entire right side of the face was captured and saved to a computer. The region of interest (ROI) on each image was defined (masked) in the same way for all images by using predefined landmarks on the face (eg, left and right corners of eye, bridge of nose, corner of mouth). The ROI was analyzed using customized software that automatically identified and quantified (1) hyperpigmented macules (spots), (2) pores, (3) rough texture, and (4) wrinkles and fine lines. Spots were defined as hyperpigmented areas on the face, either red or brown, larger than 1.5 mm^2^. The term pores was used to refer to the total dimensions of circular areas of defined tone that were smaller than 1.5 mm^2^. Texture was determined by using fractal dimension calculations^20^ that show asperity of the facial skin surface. The term wrinkles was used to refer to the total area of facial wrinkles that were longer than 5 mm and wider than 0.16 mm.

Validation to confirm imaging accuracy was performed using a mannequin head with artificial circular brown spots of known area.^19^ The error of the summed brown spot area in the cheek and lateral periorbital area was less than 5% of the actual spot sizes. Also, in an experiment using a mannequin with artificial wrinkles, the imaging accuracy of wrinkles was within 5% of the actual value.^18^

The absolute amount of each skin feature (ie, total area of spots, total area of pores, total number of textures, and total area of wrinkles) was indexed (normalized) to the size of the ROI. In this way, the severity of a skin feature could be compared from one subject to the next; subjects with large heads (and therefore large ROIs) could be compared to subjects with small heads (and correspondingly small ROIs). These normalized values are defined as hyperpigmented spot area fraction (Spot), pore area fraction (Pore), texture number fraction (Texture), and wrinkle area fraction (Wrinkle) in the Results section.

### Lifestyle factors

Information on the following lifestyle factors was collected by trained interviewers using a structured questionnaire: age, alcohol consumption status (never, former, current), smoking status (never, former, current), information about living partner (living alone or with spouse/family/others), marital status (married, widowed, separated, single), education (senior high school or higher, junior high school or lower), longest-held occupation (agriculture, forestry, fishery, self-employed outdoors, laborer, self-employed indoors, clerical, professional, housewife, other), past and current medical history of life-threatening diseases (including cancer, stroke, myocardial infarction, angina, and diabetes mellitus), the duration of daily outdoor activity (subjective assessment: very long, long, medium, short, very short), and sunscreen or foundation use (no, yes).

### Statistical analysis

The associations of lifestyle factors with the 4 skin variables (spot, pore, texture, and wrinkle) were examined. The lifestyle factors examined were age (<70 years, 70–79 years, ≥80 years), alcohol consumption status, smoking status, living partner, marital status (married, other), education, occupation (mainly outdoor work: agriculture, forestry, fishery, or self-employed outdoors; mainly indoor work: laborer, self-employed indoors, clerical, professional, housewife, or other), the duration of daily outdoor activity, and sunscreen or foundation use.

We initially performed univariate analysis between lifestyle factors and the 4 skin variables. First, scatter diagrams were used to show associations between skin condition parameters and age. Fitted lines were calculated using simple regression analysis. Analysis of variance was used for age, alcohol consumption status, smoking status (for males), and duration of daily outdoor activity. The *t* test was used for the other lifestyle factors described above. Based on this initial analysis, the following 7 lifestyle factors were included in a multivariate regression analysis: age, alcohol consumption status, smoking status, marital status, education, longest-held occupation, and sunscreen or foundation use. Living partner was not used because it was strongly correlated with marital status. Also, duration of daily outdoor activity, which was not associated with any of the skin variables in the univariate analysis, was not selected because this self-reported information was not thought to reflect accurately the amount of sun exposure.

Because the distribution of wrinkle values did not fit a normal distribution, the natural logarithm was used for the regression analysis. Thus, with regard to wrinkling, the percentage increase (increase in wrinkle length fraction, as compared to reference) was calculated and used instead of coefficients in the tables. All statistical analysis was performed using STATA 9.1 (Stata Corporation, College Station, Texas). A 2-sided *P* value of <0.05 was considered statistically significant.

## RESULTS

The basic characteristics of the subjects are shown in Table 1. Mean age (± standard deviation) was 75.3 ± 6.4 years (men, 75.2 ± 6.4; women, 75.4 ± 6.3) and the proportion of men was 42.5%. Although the participant population was younger and included more women than did the nonparticipant group, other characteristics, such as the proportion of smokers, did not differ between the participants and nonparticipants. Because only 4 female subjects were categorized as former drinkers and only 5 as former smokers, they were excluded from the analysis of drinking and smoking status.

**Table 1.** Characteristics of subjects (*n* = 802)

Variable		Value^a^		

		Total	Male	Female
Age (years)				
	<70	191 (23.8)	84 (24.6)	107 (23.2)
	70–79	410 (51.1)	176 (51.6)	234 (50.8)
	≥80	201 (25.1)	81 (23.8)	120 (26.0)
Sex				
	Male	341 (42.5)	—	—
	Female	461 (57.5)	—	—
Alcohol consumption status				
	Never	499 (64.7)	113 (35.0)	386 (86.2)
	Former	31 (4.0)	27 (8.4)	4 (0.9)
	Current	241 (31.3)	183 (56.7)	58 (13.0)
Smoking status				
	Never	611 (78.2)	179 (54.6)	432 (95.4)
	Former	65 (8.3)	60 (18.3)	5 (1.1)
	Current	105 (13.4)	89 (27.1)	16 (3.5)
Marital status				
	Married	531 (69.1)	272 (84.5)	259 (57.9)
	Other (Widowed, Separated, Single)	238 (31.0)	50 (15.5)	188 (42.1)
Education				
	Elementary or junior high school	589 (76.4)	237 (73.6)	352 (78.4)
	Senior high school or higher	182 (23.6)	85 (26.4)	97 (21.6)
Longest-held occupation				
	Outdoor^b^	457 (57.3)	205 (60.5)	252 (54.9)
	Indoor^c^	341 (42.7)	134 (39.5)	207 (45.1)
Sunscreen or foundation use				
	No	595 (74.7)	327 (96.2)	268 (58.6)
	Yes	202 (25.4)	13 (3.8)	189 (41.4)

BIS values				
	Wrinkle^d^	2.4 [1.3–4.2]	2.4 [1.3–4.6]	2.4 [1.4–3.9]
	Spot	8.7 ± 3.2	10.6^e^ ± 2.9	7.3 ± 2.6
	Pore	5.0 ± 1.5	5.9^e^ ± 1.3	4.2 ± 1.3
	Texture	3.1 ± 1.3	3.7^e^ ± 1.3	2.7 ± 1.1

^a^Values are number of subjects (%) or mean ± standard deviation.

^b^Includes agriculture, forestry, fishery, and self-employment outdoors.

^c^Includes laborer, self-employed indoors, clerical, professional, housewife, and other.

^d^Median and interquartile range are shown.

“^e^” indicates statistically significant values (*t* test (male vs female), *P* < 0.05).

**Figure 1.**
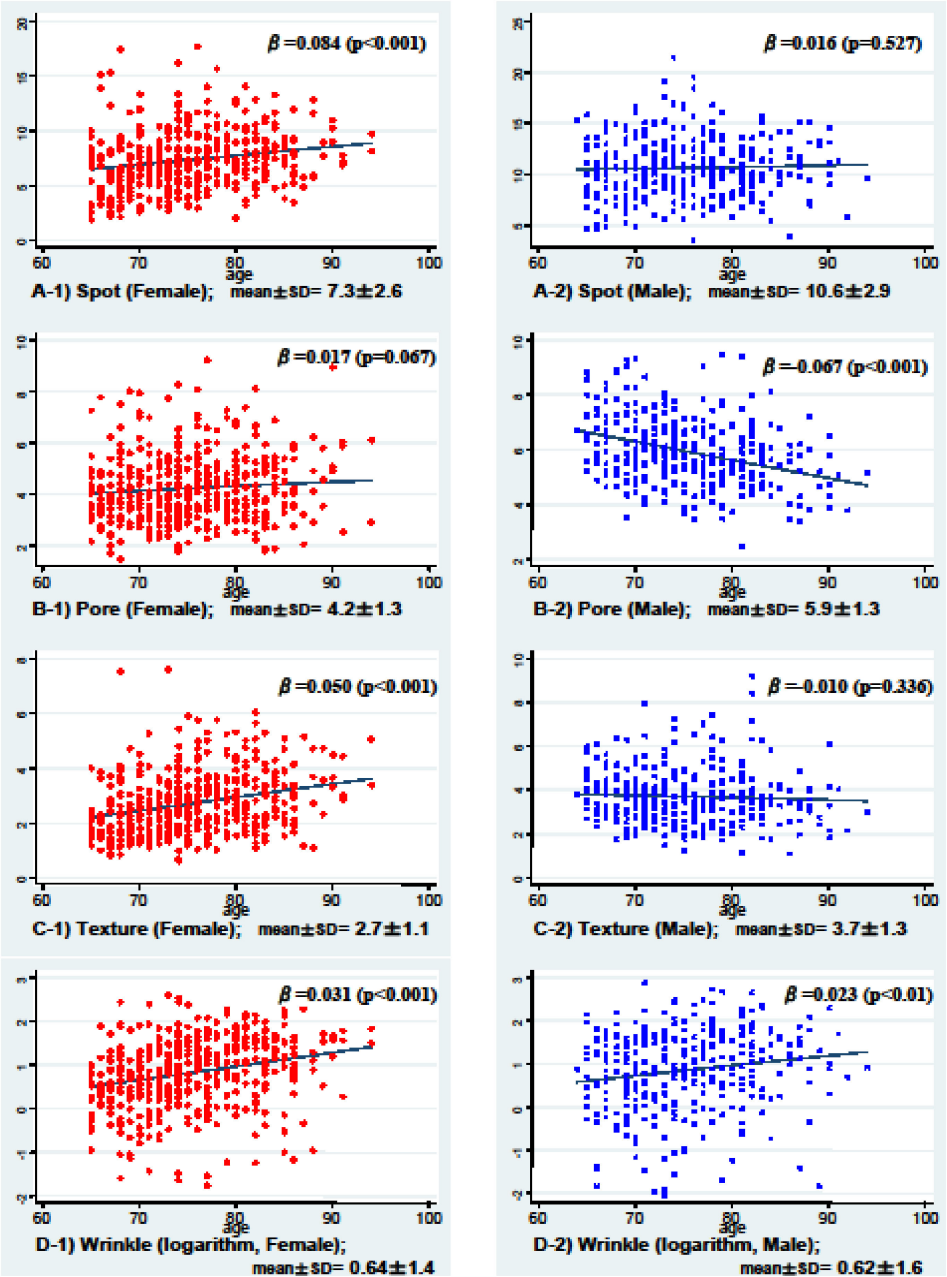
Association between skin condition parameters and age  The Y-axes of the scatter diagrams represent the values for each parameter of skin condition: A) Spot (A-1, female; A-2, male), B) Pore, C) Texture, and D) Wrinkle (logarithm). Fitted lines calculated by simple regression analysis are also displayed in the diagrams. Beta values in the diagram are the slopes of the fitted lines, and the P values indicate the results of tests of significance for the slopes

Scatter plots of the values for the skin condition parameters are shown in Figure 1. Values varied enormously, even among subjects of the same age. Spot, pore, and texture were all significantly higher in men than in women (Table 1 and Figure 1). Therefore, subsequent analysis of the men was performed separately. Figure 1 also shows the association between skin condition parameters and age. As the female subjects became older, the values for spot, texture, and wrinkle increased. Only wrinkle showed an age-related increase among male subjects.

The associations between lifestyle factors and the 4 skin condition parameters are summarized in Table 2 (a: men, b: women). Current smoking was correlated with worse skin condition in both sexes, even in mutually adjusted models. In comparison with non-smokers, the multivariate-adjusted differences (95% confidence interval) for current smokers were 1.34 (0.58–2.09) for male spot, 0.43 (0.11–0.76) for male pore, 0.46 (0.13–0.80) for male texture, 0.88 (0.20–1.56) for female pore, and 0.86 (0.29–1.42) for female texture.

**Table 2.** Skin condition indicators

a) Male									

Factors	*n*	Spot		Pore		Texture		Wrinkle	
					
Class		Crude value^a^	Adjusted difference^b^ (95%CI)	Crude value^a^	Adjusted difference^b^ (95%CI)	Crude value^a^	Adjusted difference^b^ (95%CI)	Crude value^a^	Adjusted %increase^b^ (95%CI)
Alcohol consumption status									
Never	113	10.12 ± 2.77	ref	5.78 ± 1.22	ref	3.62 ± 1.23	ref	1.79 [1.27–4.63]	ref
Former	27	10.68 ± 2.72	0.43 (−0.79, 1.64)	5.93 ± 1.40	0.10 (−0.43, 0.62)	3.82 ± 1.41	0.12 (−0.42, 0.66)	2.41 [1.40–5.14]	49.62 (−26.05, 202.72)
Current	183	10.99^d^ ± 2.95	0.82^d^ (0.14, 1.49)	6.10 ± 1.28	0.25 (−0.04, 0.54)	3.74 ± 1.28	0.09 (−0.21, 0.39)	1.75 [1.19–4.44]	4.54 (−29.44, 54.89)

Smoking status									
Never	179	10.21 ± 3.08	ref	5.75 ± 1.22	ref	3.50 ± 1.21	ref	1.81 [1.20–4.13]	ref
Former	60	10.59 ± 2.17	0.04 (−0.84, 0.92)	6.08 ± 1.30	0.20 (−0.17, 0.58)	3.91 ± 1.38	0.33 (−0.06, 0.72)	1.63 [1.14–5.54]	−24.93 (−54.82, 24.74)
Current	89	11.54^d^ ± 2.79	1.34^d^ (0.58, 2.09)	6.29^d^ ± 1.30	0.43^d^ (0.11, 0.76)	3.96^d^ ± 1.28	0.46^d^ (0.13, 0.80)	2.03 [1.33–4.87]	6.23 (−31.36, 64.42)

Marital Status									
Married	272	10.55 ± 2.94	ref	5.99 ± 1.31	ref	3.69 ± 1.27	ref	1.76 [1.19–4.52]	ref
Other	50	11.19 ± 2.74	0.54 (−0.33, 1.41)	5.79 ± 1.15	−0.15 (−0.53, 0.22)	3.63 ± 1.24	−0.11 (−0.49, 0.28)	2.02 [1.33–5.00]	12.02 (−32.30, 85.35)

Education									
Elementary or junior high school	237	10.62 ± 2.93	ref	5.86 ± 1.22	ref	3.67 ± 1.25	ref	2.07 [1.29–4.63]	ref
High school or higher	85	10.68 ± 2.89	0.32 (−0.41, 1.06)	6.22^d^ ± 1.41	0.15 (−0.17, 0.46)	3.75 ± 1.35	0.09 (−0.24, 0.41)	1.25 [0.99–4.19]	−34.81^d^ (−57.43, −0.15)

Longest-held occupation									
Outdoor	205	10.78 ± 2.81	ref	5.92 ± 1.37	ref	3.70 ± 1.28	ref	2.04 [1.29–4.83]	ref
Indoor	134	10.43 ± 3.04	−0.51 (−1.17, 0.15)	5.98 ± 1.08	−0.01 (−0.30, 0.27)	3.67 ± 1.23	'−0.04 (−0.33, 0.25)	1.71 [1.19–4.44]	−8.62 (−37.65, 33.91)

Sunscreen or foundation use									
No	327	10.73 ± 2.88	ref	5.95 ± 1.26	ref	3.72 ± 1.28	ref	1.92 [1.28–4.81]	ref
Yes	13	8.65^d^ ± 2.80	−2.14^d^ (−3.72, −0.55)	5.48 ± 0.96	−0.66 (−1.34, 0.02)	2.92^d^ ± 0.82	−0.75^d^ (−1.45, −0.05)	1.00 [1.03–2.92]	−48.75 (−79.52, 28.20)


b) Female									

Factors	*n*	Spot		Pore		Texture		Wrinkle	
					
Class		Crude value^a^	Adjusted difference^b^ (95%CI)	Crude value^a^	Adjusted difference^b^ (95%CI)	Crude value^a^	Adjusted difference^b^ (95%CI)	Crude value^a^	Adjusted % increase^b^ (95%CI)

Alcohol consumption status^c^									
Never	386	7.38 ± 2.57	ref	4.22 ± 1.27	ref	2.68 ± 1.06	ref	1.92 [1.40–3.99]	ref
Current	58	7.22 ± 2.65	0.17 (−0.58, 0.91)	4.37 ± 1.41	0.11 (−0.26, 0.48)	2.80 ± 1.17	0.20 (−0.10, 0.51)	1.64 [1.33–3.56]	−5.22 (−36.89, 42.33)

Smoking status^c^									
Never	432	7.35 ± 2.60	ref	4.20 ± 1.30	ref	2.67 ± 1.08	ref	1.89 [1.36–3.87]	ref
Current	16	7.73 ± 2.39	0.59 (−0.79, 1.97)	4.89 ± 0.99	0.88^d^ (0.20, 1.56)	3.61^d^ ± 1.36	0.86^d^ (0.29, 1.42)	3.02 [1.71–5.24]	26.64 (−40.34, 168.79)

Marital Status									
Married	259	7.22 ± 2.48	ref	4.14 ± 1.29	ref	2.61 ± 1.07	ref	1.74 [1.34–3.69]	ref
Other	188	7.54 ± 2.75	−0.01 (−0.53, 0.50)	4.35 ± 1.28	0.19 (−0.07, 0.45)	2.83^d^ ± 1.12	0.07 (−0.14, 0.28)	2.08 [1.43–4.24]	2.26 (−22.92, 35.65)

Education									
Elementary or junior high school	352	7.54 ± 2.61	ref	4.25 ± 1.30	ref	2.76 ± 1.14	ref	1.82 [1.41–3.99]	ref
High school or higher	97	6.73^d^ ± 2.40	−0.69^d^ (−1.29, −0.09)	4.15 ± 1.24	−0.08 (−0.37, 0.22)	2.50^d^ ± 0.93	−0.21 (−0.46, 0.04)	2.13 [1.32–3.75]	20.40 (−13.29, 67.18)

Longest-held occupation									
Outdoor	252	7.36 ± 2.60	ref	4.22 ± 1.24	ref	2.73 ± 1.10	ref	1.83 [1.33–4.00]	ref
Indoor	207	7.25 ± 2.60	0.16 (−0.35, 0.66)	4.22 ± 1.35	0.01 (−0.24, 0.26)	2.66 ± 1.10	0.02 (−0.19, 0.23)	2.00 [1.49–3.90]	19.76 (−9.02, 57.65)

Sunscreen or foundation use									
No	268	7.62 ± 2.49	ref	4.36 ± 1.28	ref	2.84 ± 1.10	ref	1.93 [1.43–4.02]	ref
Yes	189	6.87^d^ ± 2.70	−0.52^d^ (−1.03, 0.00)	4.01^d^ ± 1.25	−0.29^d^ (−0.55, −0.04)	2.50^d^ ± 1.08	−0.22^d^ (−0.43, −0.01)	1.92 [1.32–3.86]	6.06 (−19.72, 40.11)

^a^Values are geometric mean and interquartile range for Wrinkle, and arithmetic mean ± standard deviation for Spot, Pore, and Texture. Analysis of variance was used for univariate analysis of alcohol consumption and smoking status (for men) as a test of significance. The 2-sample *t* test was used for other variables.

^b^Differences and %increases were adjusted for age, alcohol consumption status, smoking status, marital status, education, occupation, and sunscreen or foundation use by using a multivariate regression model.

^c^Female subjects classified as former smokers (*n* = 5) or former drinkers (*n* = 4) were excluded from the analysis.

^d^indicates statistically significant values (*P* < 0.05).

Sunscreen or foundation use (a general measure of sun protection habits) was also associated with better skin condition in both sexes. In comparison with nonusers, the multivariate-adjusted differences for users were −2.14 (−3.72 to −0.55) for male spot, −0.75 (−1.45 to −0.05) for male texture, −0.52 (−1.03 to 0.00) for female spot, −0.29 (−0.55 to −0.04) for female pore, and −0.22 (−0.43 to −0.01) for female texture.

As there was no adequate indicator to represent exposure to sunlight, we used the subject’s occupation as an indicator of sunlight exposure in the analysis. However, longest-held occupation was not associated with any of the skin condition indicators. Other lifestyle factors examined in the present study also were not associated with skin condition. Although there was a tendency for education level to be associated with skin condition, this tendency was not consistent, which suggests that the results were chance findings.

## DISCUSSION

In this study, the skin condition of older adults in Japan was assessed using an objective and quantitative method (BIS). Increasing age was associated with worsening skin condition only in women. As for the lifestyle factors, smoking status and sunscreen or foundation use were associated with skin condition in both sexes.

There are 2 independent processes that influence skin aging: intrinsic and extrinsic aging.^12^^,^^21^^,^^22^ Intrinsic aging is an irreversible and inevitable process that occurs with the passage of time. It is characterized by cellular senescence and decreased proliferative capacity, a decrease in cellular DNA repair capacity, oxidative stress, and gene mutations. Extrinsic aging is the result of exposure to environmental factors, primarily ultraviolet irradiation. Cigarette smoking is another environmental factor which can affect skin aging.^3^^,^^4^^,^^12^^,^^14^^,^^15^^,^^23^ Avoiding environmental factors that accelerate skin aging is important because doing so may positively affect overall survival.^1^

BIS is an imaging system that captures standardized digital images of a subject’s face; image acquisition required only 3 to 5 minutes per person. This method was used in 2 previous studies.^24^^,^^25^ Akiba et al reported that people living in an environment with high sun exposure (Kagoshima prefecture, in southern Japan) have darker, more wrinkled skin than those living in an environment with low sun exposure (Akita prefecture, in northern Japan).^24^ The authors also maintained that wrinkle number may be a good marker of total sun exposure in life, but that hyperpigmentation was not related to lifetime hours in sunlight. We found that only facial wrinkling was associated with age, in both sexes. Their and our results suggest the same phenomena, because total sun exposure is closely related to age. In addition, Hillebrand et al reported that not only wrinkle and tone, but also hyperpigmented spots and texture, were attributable to the difference in geographic region (Kagoshima or Akita).^25^ However, the scatter diagram included in their report shows a wide distribution of the measured values (wrinkle length and spot area), especially in older participants, possibly because skin condition in this generation is obviously affected by differences in lifestyle factors. In the present study, a greater range of lifestyle factors were assessed—including smoking and methods to avoid sun exposure—than in previous studies that used BIS.

Among men, age was not associated with comprehensive skin condition in the present study. As discussed above, the effects of environmental factors accumulate with the passage of time. Therefore, the impacts of environmental factors might be more substantial than the effect of age itself in the elder male population. Indeed, the proportions of subjects with a history of smoking or outdoor employment were higher in men than in women. Interestingly, pores significantly decreased with age in men. It was reported that the number of visible pores increases with age through the age of 40, and decreases slightly thereafter. This age-related change in pore visibility mirrors that of the sebum secretion rate.^17^

Several studies reported that smoking affects skin condition,^3^^,^^4^^,^^12^^,^^14^^,^^15^^,^^23^ and a number of mechanisms have been suggested for this effect. Lahmann et al showed that smoking-induced metalloproteinase-1 might play an important role in the skin-aging effects of tobacco smoking.^26^ Metalloproteinase-1 degrades collagen, which accounts for at least 70% of the dry weight of the dermis. It has also been reported that there was a change in the microvasculature of skin exposed to the toxic products of cigarette smoke.^27^ In any case, smoking cessation is important for improving these conditions.

Although the progression of skin aging differs among ethnic groups,^17^ in the present study we observed that the same environmental factors implicated in earlier studies, such as smoking, were associated with skin condition. Although there might be some difference in efficacy, the same interventions could be applied, even among different ethnic groups.

There is no satisfactory method to measure lifetime ultraviolet exposure. However, the association between skin condition and sunscreen or foundation use that was found in the present study indicates a probable relation between sun exposure/protection and skin condition. Although we used several other variables as proxy markers of sun exposure, including longest-held occupation and subjective assessment of duration of daily outdoor activity, no associations between those variables and skin condition were observed. The use of sunscreen and foundation may indicate a subject’s overall sun avoidance and attention to skin care.

There are some methodological issues to be discussed in this study. First, as stated in the Methods and Results sections, the participant population was younger and included more women than did the nonparticipants. Because this might indicate that the participants were more active and healthier than the nonparticipants, the specific character of the participant population could influence the external validity of the study. However, the participants were not informed that the hypothesis of the study was that the BIS value is influenced by several factors, such as smoking and sunscreen use. Therefore, the selective dropout of subjects who either smoked and had a good BIS value or used sunscreen and had a worse BIS value is thought to be unlikely, ie, selection bias in the present study is improbable. Second, although the values measured by BIS are analyzed automatically using image analysis software, information bias might be possible if the technician who masks the captured images knew the background information of subjects. Thus, we blinded the image masking technician to this information. Third, prevention of the latitudinal effect is important, because lifetime UV exposure varies by latitude. In the present study, 80% of the subjects reported living in Gunma prefecture, Japan for their entire life. Therefore, environmental differences due to variations in inhabitancy were not large enough to obscure the effects of lifestyle factors such as daily habits and socioeconomic status. Lastly, although we attempted to adjust the model for various confounding factors, the possibility of confounding by unknown and unmeasured factors cannot be eliminated entirely.

In conclusion, skin condition analysis using BIS was practical and useful in the field. Older age was associated with worse skin condition among women but not men. Smoking status and sunscreen or foundation use were associated with skin condition among men and women. These findings suggest that good skin condition can be maintained to some extent by changes in modifiable lifestyle factors such as smoking and sunscreen use.

## References

[1] Rexbye H , Petersen I , Johansens M , Klitkou L , Jeune B , Christensen K Influence of environmental factors on facial ageing . Age Ageing. 2006;35:110–5 10.1093/ageing/afj031 16407433

[2] Daniell HW Smoker’s wrinkles. A study in the epidemiology of “crow’s feet” . Ann Intern Med. 1971;75:873–80 5134897 10.7326/0003-4819-75-6-873

[3] Kadunce DP , Burr R , Gress R , Kanner R , Lyon JL , Zone JJ Cigarette smoking: risk factor for premature facial wrinkling . Ann Intern Med. 1991;114:840–4 2014944 10.7326/0003-4819-114-10-840

[4] Helfrich YR , Yu L , Ofori A , Hamilton TA , Lambert J , King A , Effect of smoking on aging of photoprotected skin: evidence gathered using a new photonumeric scale . Arch Dermatol. 2007;143:397–402 10.1001/archderm.143.3.397 17372106

[5] Battistutta D , Pandeya N , Strutton GM , Fourtanier A , Tison S , Green AC Skin surface topography grading is a valid measure of skin photoaging . Photodermatol Photoimmunol Photomed. 2006;22:39–45 10.1111/j.1600-0781.2006.00194.x 16436180

[6] Sandby-Moller J , Poulsen T , Wulf HC Epidermal thickness at different body sites: relationship to age, gender, pigmentation, blood content, skin type and smoking habits . Acta Derm Venereol. 2003;83:410–3 10.1080/00015550310015419 14690333

[7] Sandby-Moller J , Thieden E , Philipsen PA , Heydenreich J , Wulf HC Skin autofluorescence as a biological UVR dosimeter . Photodermatol Photoimmunol Photomed. 2004;20:33–40 10.1111/j.1600-0781.2004.00059.x 14738531

[8] Sandby-Moller J , Thieden E , Philipsen PA , Schmidt G , Wulf HC Dermal echogenicity: a biological indicator of individual cumulative UVR exposure? Arch Dermatol Res. 2004;295:498–504 10.1007/s00403-004-0454-7 14997327

[9] Kollias N , Stamatas GN Optical non-invasive approaches to diagnosis of skin diseases . J Investig Dermatol Symp Proc. 2002;7:64–75 10.1046/j.1523-1747.2002.19635.x 12518795

[10] Chung JH Photoaging in Asians . Photodermatol Photoimmunol Photomed. 2003;19:109–21. 10.1034/j.1600-0781.2003.00027.x 12914595

[11] Nouveau-Richard S , Yang Z , Mac-Mary S , Li L , Bastien P , Tardy I , Skin ageing: a comparison between Chinese and European populations. A pilot study . J Dermatol Sci. 2005;40:187–93 10.1016/j.jdermsci.2005.06.006 16154324

[12] Leung WC , Harvey I Is skin ageing in the elderly caused by sun exposure or smoking? Br J Dermatol. 2002;147:1187–91 10.1046/j.1365-2133.2002.04991.x 12452869

[13] Malvy J , Guinot C , Preziosi P , Vaillant L , Tenenhaus M , Galan P , Epidemiologic determinants of skin photoaging: baseline data of the SU.VI.MAX. cohort . J Am Acad Dermatol. 2000;42:47–55 10.1016/S0190-9622(00)90008-2 10607319

[14] Chung JH , Lee SH , Youn CS , Park BJ , Kim KH , Park KC , Cutaneous photodamage in Koreans: influence of sex, sun exposure, smoking, and skin color . Arch Dermatol. 2001;137:1043–51 11493097

[15] Yin L , Morita A , Tsuji T Skin aging induced by ultraviolet exposure and tobacco smoking: evidence from epidemiological and molecular studies . Photodermatol Photoimmunol Photomed. 2001;17:178–83 10.1034/j.1600-0781.2001.170407.x 11499540

[16] Hillebrand GG , Levine MJ , Miyamoto K The age-dependent changes in skin condition in African Americans, Caucasians, East Asians, Indian Asians and Latinos . IFSCC Magazine. 2001;4:259–66

[17] Hillebrand GG, Levine MJ, Miyamoto K. The age-dependent changes in skin condition in ethnic populations from around the world. In: Berardesca E, Leveque J-L, Maibach HI, editors. Ethnic Hair and Skin. New York: Informa Healthcare; 2007. 105–22.

[18] Miyamoto K , Hillebrand GG The Beauty Imaging System: For the objective evaluation of skin condition . J Cosmet Sci. 2002;53:62–5

[19] Miyamoto K , Takiwaki H , Hillebrand GG , Arase S Development of a digital imaging system for objective measurement of hyperpigmented spots on the face . Skin Res Technol. 2002;8:227–35 10.1034/j.1600-0846.2002.00325.x 12423541

[20] MacAulay C , Palcic B Fractal texture features based on optical density surface area. Use in image analysis of cervical cells . Anal Quant Cytol Histol. 1990;12:394–8 2078262

[21] Uitto J Understanding premature skin aging . N Engl J Med. 1997;337:1463–5 10.1056/NEJM199711133372011 9358147

[22] Makrantonaki E , Zouboulis CC , William J Cunliffe Scientific Awards. Characteristics and pathomechanisms of endogenously aged skin . Dermatology. 2007;214:352–60 10.1159/000100890 17460411

[23] Freiman A , Bird G , Metelitsa AI , Barankin B , Lauzon GJ Cutaneous effects of smoking . J Cutan Med Surg. 2004;8:415–23 10.1007/s10227-005-0020-8 15988548

[24] Akiba S , Shinkura R , Miyamoto K , Hillebrand G , Yamaguchi N , Ichihashi M Influence of chronic UV exposure and lifestyle on facial skin photo-aging--results from a pilot study . J Epidemiol. 1999;9:S136–42 10709362 10.2188/jea.9.6sup_136

[25] Hillebrand GG , Miyamoto K , Schnell B , Ichihashi M , Shinkura R , Akiba S Quantitative evaluation of skin condition in an epidemiological survey of females living in northern versus southern Japan . J Dermatol Sci. 2001;27 Suppl 1:S42–52 10.1016/S0923-1811(01)00118-9 11514124

[26] Lahmann C , Bergemann J , Harrison G , Young AR Matrix metalloproteinase-1 and skin ageing in smokers . Lancet. 2001;357:935–6 10.1016/S0140-6736(00)04220-3 11289356

[27] Waeber B , Schaller MD , Nussberger J , Bussien JP , Hofbauer KG , Brunner HR Skin blood flow reduction induced by cigarette smoking: role of vasopressin . Am J Physiol. 1984;247:H895–901 6548876 10.1152/ajpheart.1984.247.6.H895

